# Benefits of Combining Physical Therapy with Occupational Therapy in Hip Arthroplasty

**DOI:** 10.3390/jpm11111131

**Published:** 2021-11-02

**Authors:** Florin Mihai Marcu, Nicoleta Negrut, Bogdan Uivaraseanu, Anamaria Ciubara, Vasile Valeriu Lupu, Felicia Dragan, Ancuta Lupu, Alexandru Bogdan Ciubara

**Affiliations:** 1 Faculty of Medicine and Pharmacy, University of Oradea, 410073 Oradea, Romania; mfmihai27@yahoo.com (F.M.M.); lnm_n10@yahoo.com (N.N.); uivaraseanu_bogdan@yahoo.com (B.U.); 2 Faculty of Medicine and Pharmacy, “Dunarea de Jos” University of Galati, 800008 Galati, Romania; anamburlea@yahoo.com (A.C.); bogdan.ciubara@ugal.ro (A.B.C.); 3 Faculty of General Medicine, “Grigore T. Popa” University of Medicine and Pharmacy, 700115 Iasi, Romania

**Keywords:** hip arthroplasty, rehabilitation program, modified Harris hip score

## Abstract

(1) Background: Hip arthroplasty (HA) is a surgery that replaces the damaged hip joint with an artificial implant called a hip prosthesis. The increase in life expectancy correlated with the population aging level, to which the increase in the number of prosthetic interventions among the young population is added, translates to the imperative need to analyze the quality of life beyond the immediate postoperative period. Strict adherence to an individualized rehabilitation program (IRP), and adapted to each patient, is followed by an improved quality of life. The main goal is the recovery of the patient with HA. This study was aimed to demonstrate that an IRP, represented by physical therapy associated with occupational therapy, improves the quality of life of patients with HA; (2) Methods: In this study, conducted between 2019 and 2021, 50 patients with HA were divided into two groups: study group—group A (25 subjects compliant with the IRP) and control group—group B (25 subjects, non-compliance with the IRP). To evaluate the two study groups, we monitored the evolution of the modified Harris hip score (mHHS) in both hips (arthroplasty hip (AH), contra lateral hip (CH)), for four months, respectively 30 days before the surgery (T0) and at 90 days after the surgery (T1); (3) Results: We notice significant differences in mHHS values at 90 days-T1 after surgery, both on AH in favor of subjects from group A vs. group B (*p* = 0.030) and on CH, where mHHS values were statistically higher in group A compared to group B (*p* < 0.001). The results of our study outline at T1 moment, both on the AH (*p* = 0.030) and on the CH (*p* < 0.001), the fact that mHHS values were statistically higher in patients included in group A compared to group B. In terms of the results for mHHS, comparing AH with CH, it is noted that the number of subjects who had a good or excellent mHHS result in group A versus group B is statistically significant in the case of CH (group A: 23 (92%) vs. group B: 11 (44%), *p* = 0.039); (4) Conclusions: The study reveals clear advantages of HA in both hips, both in subjects who complied with the IRP and those who did not comply; the higher therapeutic benefits of IRP are outlined at the level of CH compared to AH; in patients who comply with the IRP, the mHHS parameters that have improved in both CH and AH are pain, leaning, and shoes and socks activities; in addition, limping was reduced in CH.

## 1. Introduction

If the articular cartilage of the hip joint is severely damaged due to hip osteoarthritis or other causes, the mobility of the hip joint is progressively reduced so that the activities of daily living (ADL), walking or lifting and sitting on the chair, become painful and difficult, while putting on shoes or socks becomes a challenge [1]. As the disease progresses, the symptoms become worse: the pain also appears at rest, joint stiffness, which disappears after a few movements in the early stages of the disease, becomes more persistent and walking turns into limping.

If conservative treatment cannot stop the evolution towards the deterioration of the static and dynamic factors of the hip, hip arthroplasty (HA) [2] is recommended. HA is a standard surgical procedure that can effectively relieve pain, restore hip function and improve quality of life [3].

Although the recommendation for HA appears mainly as a therapeutic solution in the bone pathology of the elderly, the latest healthcare studies have revealed an increase in the prevalence of this pathology among young adults. This is due to the fact that in the last few years the population started eating unhealthily which predisposes to obesity, due to the professional activities that strain the hip joint excessively, such as prolonged sitting or driving and last but not least, due to sedentary lifestyle [4,5].

The recommendation for HA has a significant social-economic cost, therefore patients who due to structural deterioration of the hip joint are unable to get some rest at night, either cannot walk more than a few hundred meters, cannot perform their ADLs, or both, thus losing their autonomy. In the case of people currently employed, retirement or at best, professional reorientation must be taken into consideration. As regards retired people, they will restrict their daily activities and will need help in performing them. These social aspects involve significant financial costs: job loss, family involvement and appropriate social assistance. To these we add the expenses generated by the extremely complex prolonged drug treatment: analgesic, anti-inflammatory, gastric protection and antihypertensive, if needed [6].

Mainly, the role of the hip prosthesis is to totally or partially solve arthralgia, vascular deficiency of the hip, changes in pressure coefficients, instability and functionality of the hip joint. The patient with HA becomes a chronic patient and the proper preoperative and postoperative management becomes the therapeutic conduct of choice [7].

Generally, post-HA complications are rare, however, in the presence of chronic diseases, there is a higher risk of complication onset which can extend or limit the recovery period [8]. Among the possible complications associated with HA surgery, we mention infections, venous thrombosis, limb length inequality, prosthetic dislocation, paralysis of the internal or external popliteal sciatic nerve and loss of cementation or wear of the implant. Complications associated with HA can also occur due to falls, which is why this should be avoided, especially in the first weeks after the surgery. We must mention that too much exercise and being overweight accelerate wear and tear and can lead to implant damage and pain. Another complication that can occur refers to the onset or amplification of emotional, cognitive and neuropsychological disorders. In most situations, psychological assistance provided to the patient will help him/her to overcome the times of crisis [9].

In most cases, following HA, pain disappears and the quality of life is improved considerably. The result of the surgery depends on a series of factors, among which we note: the preoperative preparation, the surgeon’s ability, the patient’s general condition, the patient’s compliance and how rigorously the patient complies with the individualized rehabilitation program (IRP). The patient’s preoperative preparation involves performing a specific physical therapy program (PTP), which should begin immediately before surgery, aiming to increase the stability of the joint muscles on which prosthesis is to be fitted, thus ensuring a rapid and complete postoperative recovery of the patient [10]. IRP mainly includes PTP and occupational therapy (OT), which patients should continue after discharge [11]. In OT for HA patients, improving the performance of activities of daily living (ADL) is a central goal of the IRP [12]. Achieving this goal is essential for increasing individual independence, thereby improving the quality of life (QOL) for HA patients [13].

The literature emphasizes that, even 6–12 months after HA, functional limitations and reduced muscle strength still exist, requiring adherence to a rehabilitation protocol. This includes both preoperative and postoperative PTP and postoperative OT. According to studies, patients who do not do the IRP are dependent on walking aids [14,15].

At present, a comprehensive and integrated preoperative and postoperative rehabilitation plan is not applied. The aim of this study was to underline the effectiveness of IRP in the treatment of patients with HA.

## 2. Materials and Methods

A total of 50 patients were involved in the study. They were admitted to the Emergency Clinical County Hospital of Oradea, Romania and gave their written consent to take part in the study, based on subjective and objective anamnesis criteria. Our study was conducted over two years between 2019 and 2021. The inclusion criteria in the clinical study comprised patients hospitalized for hip prosthesis with a body mass index of up to 35 and who agreed to participate.

The exclusion criteria used were significant comorbidities that prevented the implementation of recovery programs, psychiatric disorders, malignant tumors, refusal to participate, and a body mass index above 35. For a more consistent and relevant evaluation between the two groups of subjects, patients with post arthroplasty complications were not included in the study.

Within the hip post-arthroplasty rehabilitation program, physical therapy associated with OT has a very important role in improving the patient’s quality of life. The usefulness of a PTP before surgery is mainly justified by the following objectives:

- the prevention of the muscle atrophy process after immobilization;
- the prophylaxis of osteoporosis onset;
- maintaining muscle tone;
- shortening the time required for rehabilitation [16,17].

Individualized and adapted PTP should be performed under the supervision of a physiotherapist who monitors the achievement of the proposed objectives. Immediately after the surgery, in order to maintain balance, it is recommended to use a crutch or a walking frame or to lean on another person. For at least six weeks after the surgery, it is recommended for patients: to avoid crossing their legs, not to bend the hip more than 90 degrees, not to force the internal or external rotation of the hip and to sleep with a pillow between the legs. PTP is focused on:

- optimizing breathing;
- improving muscle strength and joint mobility in pain-free circumstances or low pain intensity;
- improving stability in orthostatism and walking;
- gait re-education in the context of correct posture, meeting the loading time recommended by the orthopedic specialist [10,18].

Within OT, in order to perform most ADLs (cooking, intimate hygiene, shopping), assistance is needed initially, and later adaptive changes to the patient’s home both in order to perform the activities and to avoid falls. Activities with beneficial impact after total hip arthroplasty include walking, swimming, golf, cycling and dancing [11]. According to specialized studies, the performance of physical activities that generate increased stress on the hip joint such as jumping, sprinting or rapid changes of direction should be avoided; these types of movements are found in football, handball, hockey, tennis or martial arts, such as karate, judo and wrestling [19].

The recommendation to comply with IRP after discharge was not met by all patients, for subjective reasons of medical discipline. Depending on this aspect, two groups of subjects were organized: group A, comprising 25 subjects who have undergone a postoperative recovery program and group B, with 25 subjects who did not undergo or comply with a postoperative recovery program.

To underline the efficacy of IRP in patients with HA, we monitored the evolution of the modified Harris hip score (mHHS) within the two study groups, in both hips for 4 months, respectively 30 days before surgery (T0) and at 90 days after the surgery (T1). Thus, mHHS is a valid and reliable tool for assessing functional outcome after total hip replacement, with a positive correlation with the standard Harris hip score [20]. Consequently, mHHS is a reliable and valid tool for the functional evaluation of patients with HA [21]. The interpretation of the result using mHHS was as follows: <70 = poor result, 70–79 = fair result, 80–89 = good result and >90 = excellent result.

**Modified Harris hip score** [22]

**Pain:**

- no pain/can be ignored (44 points)
- slight, occasional, no compromise in activities (40 points)
- mild, no effect on ordinary activities, rarely moderate pain after unusual activities, uses aspirin (30 points)
- moderate, tolerable, causes some limitations in ordinary activities/work, may require pain medication occasionally that is stronger than aspirin (20 points)
- marked with serious limitations of activities (10 points)
- totally disabled, bedridden patient (0 points)

**Function**

***gait limp***

- none (11 points)
- slight (8 points)
- moderate (5 points)
- severe (0 points)
- unable to walk (0 points)

***support***

- none (11 points)
- cane for long walks (7 points)
- cane, full time (5 points)
- crutch (4 points)
- 2 canes (2 points)
- 2 crutches (1 point)
- unable to walk (0 points)

***walked distance***

- unlimited (11 points)
- can walk 1 mile (8 points)
- can walk ½ mile (5 points)
- indoors only (2 points)
- from bed to chair (0 points)

**Functional activities**

***stairs***

- normally without banister (4 points)
- normally with banister (2 points)
- uses the stairs in any manner (1 point)
- not able to use the stairs (0 points)

***socks/shoes***

- with ease (4 points)
- with difficulty (2 points)
- unable (0 points)

***sitting***

- comfortable on any chair, 1 h (5 points)
- on a highchair, ½ h (3 points)
- unable to sit on any chair (0 points)

***public transport***

- able to use public transportation (1)
- unable to use public transportation (0).

### Statistical Analysis

The statistical analysis was carried out with Statistical Package for the Social Sciences, Version 26 (IBM Corp., Armonk, NY, USA). 

The design of experiment (DOE) assumed three statistical factors: the first factor was the hip status with two levels: arthroplasty hip (AH) and contra lateral hip (CH). The second factor was the treatment: with rehabilitation—group A and without—group B. The third factor was the time between diagnosis and surgery: 30 days before surgery (T0), and 90 days after the surgery (T1). The mHHS values were gathered into 8 groups: AH_B_T0, AH_B_T1, CH_B_T0, CH_B_T1, AH_A_T0, AH_A_T1, CH_A_T0 and CH_A_T1, with N = 25 patients in each group. All these values were tested for normal distribution with D’Agostino and Pearson omnibus normality test, Shapiro–Wilk normality test and Kolmogorov–Smirnov normality test.

The data were presented in the form of tables and graphs and the results were interpreted in numerical, percentage form. The *p* values were generated using parametric tests, Student and chi-square tests, applied for the arithmetic average value, the standard deviation within the two groups, group A with recovery program versus group B without recovery program, by comparing the mHHS values. A value below 0.05 was accepted as statistically significant.

This study was conducted according to the guidelines of the Declaration of Helsinki and it was approved by the Ethical Commission and the Ethical Council of the Emergency Clinical County Hospital of Oradea, Romania (registration No. 25572, 25571/24.10.2019).

## 3. Results

The normality tests were performed for mHHS, DSI (diagnosis–surgery interval) and BMI (body mass index). Normality test results (*p* = 0.05) for mHHS show that all 8 patient groups have normal distribution, as it can see in Table 1. This fact prescribes parametric statistical test use for mHHS mean values comparisons.

The DSI and BMI parameters were tested for better description of the treatment factor levels, with rehabilitation, group A, and without rehabilitation, group B. As can be noticed from Table 2, these parameters prescribe no statistical difference of the coefficient of variation and distribution type. This result confirms that the DOE was done with consistent data values. 

To describe the two groups, we referred to gender, background, age, diagnosis-surgery interval and body mass index. The two subject groups, group A and group B, did not present statistically significant different characteristics (Table 3 and Table 4).

By comparing the initial values, we sought to observe if there are statistically significant differences between the two studied groups.

**Figure 1:**
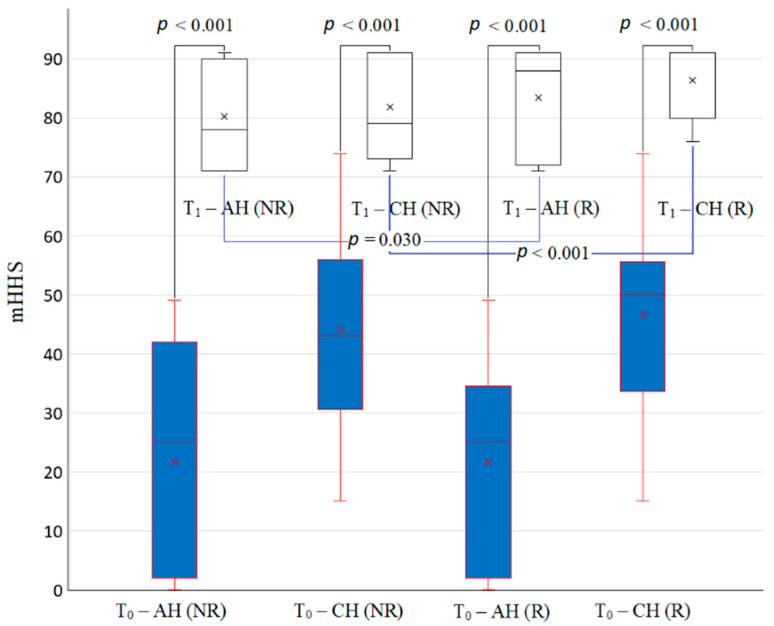
The evolution of the modified mHHS during the study. Legend: mHHS—modified Harris hip score; group A—group with recovery, group B—group without recovery, AH—arthroplasty hip, CH—contra lateral hip, *p* values—statistical significance (*t*-test), T_0_—before surgery, T_1_—90 days after the surgery.

Favorable results were obtained after the surgery, both for group B and for group A. The values of mHHS, 30 days (T0) before the surgery were statistically significantly lower than those obtained after 90 days from the surgery (T1), both for AH and for CH in groups A and B (AH: (21.52 ± 18.74 vs. 80.16 ± 8.62 (group B) and 21.6 ± 18.00 vs. 83.4 ± 8.90 (group A)); CH: (44.04 ± 16.33 vs. 81.48 ± 8.39 (group B) and 46.44 ± 15.86 vs. 86.6 ± 5.70 (group A))) (Figure 1).

We note significant differences in mHHS values at 90 days (T1) after surgery, both on AH in favor of subjects from group A vs. group B (*p* = 0.030) and on CH, where mHHS values were statistically higher in group A compared to group B (*p* < 0.001) (Figure 1).

**Figure 2:**
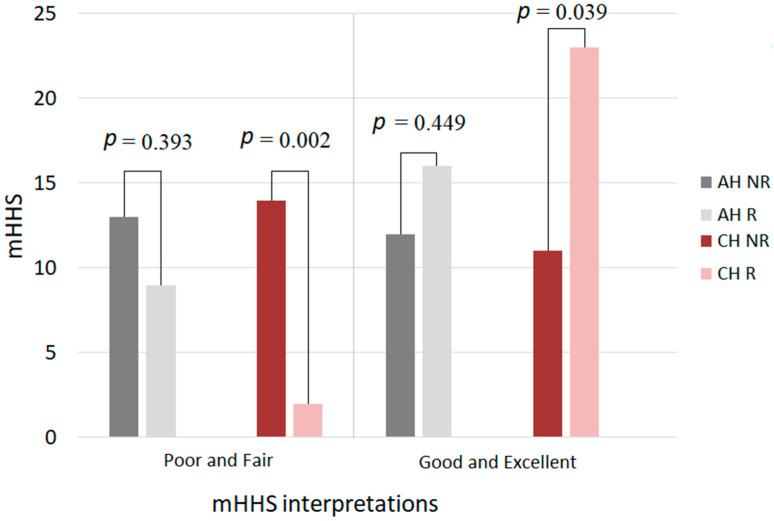
Comparative interpretation of mHHS between the studied groups at the end of the study. Legend: mHHS—modified Harris hip score; group A—group with recovery, group B—group without recovery, AH—arthroplasty hip, CH—contra lateral hip, *p* values—statistical significance (chi-square test).

As for CH, we found that the results for mHHS in group A are good and excellent, compared to group B (group A: 23 (92%) vs. group B: 11 (44%), *p* = 0.039). Poor and fair results were found mostly in group B (group A: 2 (8%) vs. group B: 14 (56%), *p* = 0.002); the difference was statistically significant (Figure 2).

Regarding the AH, the number of subjects who had good, excellent or poor and fair mHHS result were not significantly different between groups A and B (poor and fair—group B: 13 (52%) vs. group A: 9 (36%), *p* = 0.393; good and excellent—group B: 12 (48%) vs. group A: 16 (64%), *p* = 0.449) (Figure 2).

At the AH level, comparing group A with group B at the end of the study (T1), in terms of each parameter of the mHHS, a statistically significant improvement of the parameter “pain, support and shoes and socks activities” was noted (Table 5). 

At the CH level, comparing group A with group B at the end of the study (T1), a significant improvement was noticed in the following parameters of mHHS: pain, limping, leaning and shoes and socks activities (Table 6).

## 4. Discussion

According to specialized studies, HA has proven to be successful in the treatment of functionally decompensated hip osteoarthritis, leading to pain relief and improved functionality. Patients, who have undergone HA, in terms of the clinical image-quality of life relationship, went through the following situations that need to be managed: reduced quality of life due to pain and disabilities during the preoperative period, increased quality of life due to no restrictions during the immediate postoperative period and individual adaptation of each patient to the prosthesis [23,24].

The results of our study, which show that subjects in both groups, with or without a recovery program, have higher mHHS values at T1 stage versus T0 stage, confirm the above-mentioned.

According to Luigi Zagra, the comparative financial costs of HA versus the conservative treatment plus secondary complications to pathology and treatment, evaluated in terms of the patient’s quality of life, show that HA is an efficient and safe procedure [25].

The hip replacement procedure is increasingly indicated in adults who are currently active employees and who, in most cases, want to return to professional activities and sometimes to sports activities. The argument for suggesting HA to adults who are active employees is the fact that the majority of patients with HA return to professional and sports activities within a period ranging between 17 and 28 weeks after the surgery, depending on compliance with the IRP [26].

In patients with HA, adherence to a long-term IRP that includes specific physical therapy exercises performed preoperatively and postoperatively, associated with specific occupational therapy activities, including the use of adaptive devices (canes, crutches, walking frames) increases the patient’s quality of life due to relief of symptoms (pain relief, increased strength of the muscles that cover the joint) and increased autonomy and improvement of joint functionality [27,28]. The compliance with a program of specific physiotherapy exercises shortens the hospitalization period and accelerates the recovery process [29]. After 4 months of IRP, at the T1 stage, it is noted that the values of mHHS, both on AH (*p* = 0.030) and on CH (*p* < 0.001), are superior in favor of the subjects from group A vs. group B; this aspect clearly underlines the positive impact of the IRP in terms of the recovery program.

The benefits of IRP are materialized only if this program is continued for a long period of time and it is also complied with at the patient’s home [30]. The results of our study confirm that HA represents only half of the difficult struggle in order to obtain a new and functional hip joint, while functional rehabilitation, performed with the help of an IRP, represents the other half; this aspect is also revealed in specialty studies [31,32]. The benefits of IRP can be seen at the level of both hips, both at the level of AH and at the level of CH, however it is noticed that the clearest benefits of IRP are identified at the level of CH. This aspect is explained by the fact that HA clearly increases the functionality of the AH and IRP completes the rehabilitation process. Instead, in terms of CH, as shown in other specialty studies [33,34], the benefit of IRP is obvious by comparing the results of the two studied groups.

## 5. Conclusions

The results of our study underline the clear advantages of HA in both hips, both in subjects who have met the IRP and those who have not met the IRP; it reflects the benefits of IRP in the case of patients with HA compared to those who do not comply with PTP and OT. The top performance of IRP is highlighted at the level of CH compared to AH. In patients who have complied with IRP, the mHHS parameters that have improved in both CH and AH are pain, leaning and shoes and socks activities; in addition, limping was reduced in CH.

In particular, it should be noted that complying with IRP determines more obvious benefits at the level of CH, compared to AH whose functionality is already clearly improved after HA. The particularity of our study comes from the benefits of associating the physical therapy program with the activities characteristic of occupational therapy in patients with hip arthroplasty.

## Figures and Tables

**Table 1:** Normality test results of the mHHS, for all studied sample groups (hip (AH/CH), treatment (B/A), time (T0/T1)).

mHHS	AH_B_T0	AH_B_T1	CH_B_T0	CH_B_T1	AH_A_T0	AH_A_T1	CH_A_T0	CH_A_T1
**Mean**	**61.52**	**80.16**	**74.04**	**81.48**	**71.6**	**83.4**	**66.44**	**76.6**
Std. Deviation	8.75	8.63	6.33	8.397	8.01	8.902	5.86	5.708
Std. Error of Mean	2.749	1.726	1.267	1.679	1.601	1.78	1.172	1.842
Lower 95% CI of mean	63.78	76.6	77.3	78.01	74.17	79.73	79.89	84.24
Upper 95% CI of mean	79.26	73.72	70.78	74.95	69.03	87.07	72.99	78.96
**D’Agostino and Pearson omnibus normality test**								
K2	0.2223	0.2948	0.494	0.2387	0.7358	0.1762	0.2194	0.8226
*p* value	**0.6891**	**0.5678**	**0.7812**	**0.6012**	**0.5252**	**0.9121**	**0.8961**	**0.7164**
Passed normality test (alpha = 0.05)?	Yes	Yes	Yes	Yes	Yes	Yes	Yes	Yes
*p* value summary	ns	ns	ns	ns	ns	ns	ns	ns
**Shapiro–Wilk normality test**								
W	0.8406	0.7971	0.9597	0.8122	0.8774	0.9291	0.9667	0.7174
*p* value	**0.5012**	**0.6102**	**0.4076**	**0.7904**	**0.8661**	**0.7701**	**0.562**	**0.6745**
Passed normality test (alpha = 0.05)?	Yes	Yes	Yes	Yes	Yes	Yes	Yes	Yes
*p* value summary	ns	ns	ns	ns	ns	ns	ns	ns
**KS normality test**								
KS distance	0.2111	0.1366	0.1303	0.1315	0.2218	0.2833	0.1134	0.1796
*p* value	**0.25**	**0.2**	**0.2**	**0.3**	**0.26**	**0.3**	**0.2**	**0.3**
Passed normality test (alpha = 0.05)?	Yes	Yes	**Yes**	Yes	Yes	Yes	**Yes**	**Yes**
*p* value summary	ns	ns	ns	ns	ns	ns	ns	ns
**Coefficient of variation (%)**	**37.11**	**40.77**	**37.09**	**40.31**	**33.37**	**35.67**	**34.16**	**36.59**

**Table 2:** Normality test results of DSI and of BMI for group A and group B.

	DSI_A	BMI_A	DSI_B	BMI_B
**Mean**	**11.52**	**27.15**	**12.12**	**27.87**
Std. Deviation	3.798	3.337	3.8	3.371
Std. Error of Mean	0.7596	0.6673	0.7601	0.6741
Lower 95% CI of mean	9.952	25.77	10.55	26.48
Upper 95% CI of mean	13.09	28.53	13.69	29.26
**D’Agostino and Pearson omnibus normality test**				
K2	5.785	0.1668	2.978	0.342
*p* value	**0.0554**	**0.92**	**0.2256**	**0.8428**
Passed normality test (alpha = 0.05)?	Yes	Yes	Yes	Yes
*p* value summary	ns	ns	ns	ns
**Shapiro–Wilk normality test**				
W	0.8892	0.9822	0.9066	0.9781
*p* value	**0.0107**	**0.9253**	**0.0256**	**0.8452**
Passed normality test (alpha = 0.05)?	No	Yes	No	Yes
*p* value summary	*	ns	*	ns
**KS normality test**				
KS distance	0.2097	0.1182	0.1915	0.09876
*p* value	**0.006**	**0.2**	**0.0187**	**0.2**
Passed normality test (alpha = 0.05)?	No	Yes	No	Yes
*p* value summary	**	ns	*	ns
**Coefficient of variation (%)**	**32.97**	**12.29**	**31.36**	**12.09**

*—statistical significance level (more stars higher significance level).

**Table 3:** Patient distribution in the two groups.

Parameter	Group A	Group B	*p*	OR	95% CI
Age, M, SD	58.64 ± 6.83	59 ± 5.39	0.837 *	–	–
Female, N (%)	18 (72%)	19 (76%)	0.500 **	0.81	0.22–2.88
Urban area, N (%)	22 (88%)	18 (68%)	0.144 **	2.85	0.64–12.64
DSI, M, SD	11.52 ± 3.80	12 ± 3.80	0.657 *	–	–
BMI, M, SD	30.53 ± 2.16	30 ± 1.80	0.350 *	–	–
mHHS					
AH, M, SD	21.6 ± 18.00	21.52 ± 18.74	0.987 *	–	–
CH, M, SD	46.44 ± 15.86	44.04 ± 16.33	0.600 *		–

Legend: DSI—diagnosis-surgery interval (months), BMI—body mass index, mHHS—modified Harris hip score, AH—arthroplasty hip, CH—contra lateral hip, group A—group with recovery, group B—group without recovery, M—average value, SD—standard deviation, OR—odds ratios, CI—confidence intervals, *p* values—statistical significance (*—*t*-test, **—chi-square test).

**Table 4:** The initial values of mHHS in the two groups.

mHHS	Group A	Group B	*p*
AH			–
poor result, N, %	25 (100%)	25 (100%)	1 *
fair result, N, %	0 (0%)	0 (0%)	–
good result, N, %	0 (0%)	0 (0%)	–
excellent result, N, %	0 (0%)	0 (0%)	–
CH			
poor result, N, %	23 (92%)	23 (92%)	1 *
fair result, N, %	2 (8%)	2 (8%)	1 *
good result, N, %	0 (0%)	0 (0%)	–
excellent result, N, %	0 (0%)	0 (0%)	–

Legend: mHHS—modified Harris hip score, AH—arthroplasty hip CH –contra lateral hip, group A—group with recovery, group B—group without recovery, *p* values—statistical significance (*—chi-square test).

**Table 5:** The values of the mHHS parameters at the AH level, at the end of the study.

Parameter for AH	Group B	Group A	*p*
Pain, M, SD	41.12 ± 1.83	42.08 ± 2.03	0.027
Function, M, SD			
Limp	9.44 ± 1.52	9.8 ± 1.5	0.092
Distance walked Support	9.56 ± 1.52	9.8 ± 1.5	0.212
	8.76 ± 2.02	9.56 ± 1.95	0.028
Activities, M, SD			
Stairs	3.04 ± 1.01	3.28 ± 0.97	0.092
Shoes and socks	2.88 ± 1.01	3.44 ± 0.91	0.008
Sitting	4.36 ± 0.95	4.44 ± 0.91	0.331
Public transportation	1 ± 0	1 ± 0	–

Legend: mHHS—modified Harris hip score; AH—arthroplasty hip, CH—contralateral hip, group A—group with recovery, group B—group without recovery, M—average value, SD—standard deviation, *p* values—statistical significance (*t*-test).

**Table 6:** The values of the mHHS parameters at the CH level, at the end of the study.

Parameter for CH	Group B	Group A	*p*
Pain, M, SD	41.76 ± 2.03	43.52 ± 1.33	<0.001
Function, M, SD			
Limp	9.56 ±1.53	10.52 ± 1.12	0.004
Distance walked Support	9.56 ± 1.53	9.8 ± 1.50	0.212
	8.76 ± 2.03	9.56 ± 1.96	0.028
Activities, M, SD			
Stairs	2.96 ± 1.02	3.28 ± 0.98	0.051
Shoes and socks	3.6 ± 0.82	4 ± 0	0.010
Sitting	4.28 ± 0.98	4.44 ± 0.92	0.212
Public transportation	1 ± 0	1 ± 0	–

Legend: mHHS—modified Harris hip score; AH—arthroplasty hip, CH—contralateral hip, group A—group with recovery, group B—group without recovery, M—average value, SD—standard deviation, *p* values—statistical significance (*t*-test).

## Data Availability

The datasets either used, analyzed, or both, during the current study are available from the corresponding authors on reasonable requests.

## References

[1] Kasnakova P., Ivanova S., Ivanov K., Petkova-Gueorguieva E., Gueorguiev S., Madzharov V., Mihaylova A., Petleshkova P. (2018). Conservative therapy options for the treatment of coxarthrosis in the early stage of the condition. Biomed. Res..

[2] Nazal M., Parsa A., Martin S.D. (2019). Arthroscopic Diagnosis and Treatment of Chronic Hip Pain After Total Hip Arthroplasty and the Role of Anterior Capsule Disruption in Iliopsoas Tendinopathy. Orthop. J. Sports Med..

[3] Park K.K., Tsai T.-Y., Dimitriou D., Kwon Y.-M. (2015). Three-dimensional in vivo difference between native acetabular version and acetabular component version influences iliopsoas impingement after total hip arthroplasty. Int. Orthop..

[4] Skyttä E.T., Jarkko L., Antti E., Huhtala H., Ville R. (2011). Increasing incidence of hip arthroplasty for primary osteoarthritis in 30- to 59-year-old patients A population based study from the Finnish Arthroplasty Register. Acta Orthop..

[5] Daras M., Macaulay W. (2009). Total hip arthroplasty in young patients with osteoarthritis. Am. J. Orthop..

[6] Koenig L., Zhang Q., Austin M.S., Demiralp B., Fehring T.K., Feng C., Mather R.C.I.I.I., Nguyen J.T., Saavoss A., Springer B.D. (2016). Estimating the Societal Benefits of THA After Accounting for Work Status and Productivity: A Markov Model Approach. Clin. Orthop. Relat. Res..

[7] Liu X.-W., Zi Y., Xiang L.-B., Wang Y. (2015). Total hip arthroplasty: A review of advances, advantages and limitations. Int. J. Clin. Exp. Med..

[8] Boisgard S., Descamps S., Bouillet B. (2013). Complex primary total hip arthroplasty. Orthop. Traumatol. Surg. Res..

[9] Hwang S.K. (2014). Experience of Complications of Hip Arthroplasty. Hip Pelvis.

[10] Madara K.C., Marmon A., Aljehani M., Hunter-Giordano A., Zeni J.J., Raisis L. (2019). Progressive rehabilitation after total hip arthoplasty: A pilot and feasibility study. Int. J. Sports Phys. Ther..

[11] Krastanova M.S., Ilieva E.M., Vacheva D.E. (2017). Rehabilitation of Patients with Hip Joint Arthroplasty (Late Post-surgery Period—Hospital Rehabilitation). Folia Med..

[12] Barnsley L., Barnsley L., Page R. (2015). Are Hip Precautions Necessary Post Total Hip Arthroplasty? A Systematic Review. Geriatr. Orthop. Surg. Rehabil..

[13] Tetreault M.W., Akram F., Li J., Nam D., Gerlinger T.L., Della Valle C.J., Levine B.R. (2020). Are Postoperative Hip Precautions Necessary After Primary Total Hip Arthroplasty Using a Posterior Approach? Preliminary Results of a Prospective Randomized Trial. J. Arthroplast..

[14] Judd D.L., Dennis D.A., Thomas A.C., Wolfe P., Dayton M.R., Stevens-Lapsley J.E. (2014). Muscle strength and functional recovery during the first year after THA. Clin. Orthop. Relat. Res..

[15] Ninomiya KHirakawa K., Ikeda T., Nakura N., Suzuki K. (2018). Patients 10 years after total hip arthroplasty have the deficits in functional performance, physical activity, and high fall rate compared to healthy adults. Phys. Ther. Res..

[16] Czyżewska A., Glinkowski W.M., Walesiak K., Krawczak K., Cabaj D., Górecki A. (2014). Effects of preoperative physiotherapy in hip osteoarthritis patients awaiting total hip replacement. Arch. Med. Sci..

[17] Coudeyre E., Jardin C., Givron P., Ribinik P., Revel M., Rannou F. (2007). Could preoperative rehabilitation modify postoperative outcomes after total hip and knee arthroplasty? Elaboration of French clinical practice guidelines. Ann. Réadapt. Méd. Phys..

[18] Monaco M.D., Vallero F., Tappero R., Cavanna A. (2009). Rehabilitation after total hip arthroplasty: A systematic review of controlled trials on physical exercise programs. Eur. J. Phys. Rehabil. Med..

[19] Vogel L.A., Carotenuto G., Basti J.J., Levine W.N. (2011). Physical Activity after Total Joint Arthroplasty. Sports Health.

[20] Kumar P., Sen R., Aggarwal S., Agarwal S., Rajnish R.K. (2019). Reliability of Modified Harris Hip Score as a tool for outcome evaluation of Total Hip Replacements in Indian population. J. Clin. Orthop. Trauma..

[21] Gupta L., Lal M., Aggarwal V., Rathor L.P. (2018). Assessing functional outcome using modified Harris hip score in patients undergoing total hip replacement. Int. J. Orthop. Sci..

[22] Modified Harris Hip Score. https://www.losangelessportssurgeon.com/pdf/modified-harris-hip-score.pdf.

[23] Okafor L., Chen A.F. (2019). Patient satisfaction and total hip arthroplasty: A review. Arthroplasty.

[24] Graham B., Green A., James M., Katz J., Swiontkowski M. (2015). Measuring patient satisfaction inorthopaedic surgery. J. Bone Jt. Surg..

[25] Zagra L. (2017). Advances in hip arthroplasty surgery: What is justified?. EFORT Open Rev..

[26] Hoorntje A., Janssen K.Y., Bolder S.B.T., Koenraadt K.L.M., Daams J.G., Blankevoort L., Kerkhoffs G.M.M.J., Kuijer P.P.F.M. (2018). The Effect of Total Hip Arthroplasty on Sports and Work Participation: A Systematic Review and Meta-Analysis. Sports Med..

[27] Mark-Christensen T., Thorborg K., Kallemose T., Bandholm T. (2021). Physical rehabilitation versus no physical rehabilitation after total hip and knee arthroplasties: Protocol for a pragmatic, randomized, controlled, superiority trial (The DRAW1 trial). F1000Research.

[28] Bozorgi A.A.J.B., Ghamkhar L., Kahlaee A.H., Sabouri H. (2015). The Effectiveness of Occupational Therapy Supervised Usage of Adaptive Devices on Functional Outcomes and Independence after Total Hip Replacement in Iranian Elderly: A Randomized Controlled Trial. Occup. Ther. Int..

[29] Smith T.O., McCabe C., Lister S., Christie S.P., Cross J. (2012). Rehabilitation implications during the development of the Norwich Enhanced Recovery Programme (NERP) for patients following total knee and total hip arthroplasty. Orthop. Traumatol. Surg. Res..

[30] Harris J., Orpen N. (2010). Patients’ Perceptions of Preoperative Home-Based Occupational Therapy and/or Physiotherapy Interventions Prior to Total Hip Replacement. Br. J. Occup. Ther..

[31] Paunescu F., Didilescu A., Antonescu D.M. (2014). Does Physiotherapy Contribute to the Improvement of Functional Results and of Quality of Life after Primary Total Hip Arthroplasty?. Maedica.

[32] Păunescu F., Didilescu A., Antonescu D.M. (2013). Factors that may influence the functional outcome after primary total hip arthroplasty. Clujul Med..

[33] Papalia R., Campi S., Vorini F., Zampogna B., Vasta S., Papalia G., Fossati C., Torre G., Denaro V. (2020). The Role of Physical Activity and Rehabilitation Following Hip and Knee Arthroplasty in the Elderly. J. Clin. Med..

[34] Wu J.Q., Mao L.B., Wu J. (2019). Efficacy of exercise for improving functional outcomes for patients undergoing total hip arthroplasty: A meta-analysis. Medicine.

